# Modulation of Plant MicroRNA Expression: Its Potential Usability in Wheat (*Triticum aestivum L*.) Improvement

**DOI:** 10.2174/0113892029264886231016050547

**Published:** 2023-12-12

**Authors:** Louie Cris Lopos, Urbashi Panthi, Igor Kovalchuk, Andriy Bilichak

**Affiliations:** 1 Morden Research and Development Centre, Agriculture and Agri-Food Canada, Morden, MB R6M 1Y5, Canada;; 2 Department of Biological Sciences, University of Lethbridge, Lethbridge, AB T1K 3M4, Canada

**Keywords:** microRNAs, wheat improvement, yield, abiotic stress, somatic embryogenesis, thermotolerance

## Abstract

Wheat, a crucial crop for the pursuit of food security, is faced with a plateauing yield projected to fall short of meeting the demands of the exponentially increasing human population. To raise global wheat productivity levels, strong efforts must be made to overcome the problems of (1) climate change-induced heat and drought stress and (2) the genotype-dependent amenability of wheat to tissue culture, which limits the success of recovering genetically engineered plants, especially in elite cultivars. Unfortunately, the mainstream approach of genetically engineering plant protein-coding genes may not be effective in solving these problems as it is difficult to map, annotate, functionally verify, and modulate all existing homeologs and paralogs within wheat’s large, complex, allohexaploid genome. Additionally, the quantitative, multi-genic nature of most agronomically important traits furthers the complications faced by this approach. miRNAs are small, noncoding RNAs (sncRNAs) that repress gene expression at the post-transcriptional level, regulating various aspects of plant growth and development. They are gaining popularity as alternative targets of genetic engineering efforts for crop improvement due to their (1) highly conserved nature, which facilitates reasonable prediction of their gene targets and phenotypic effects under different expression levels, and (2) the capacity to target multiple genes simultaneously, making them suitable for enhancing complex and multigenic agronomic traits. In this mini-review, we will discuss the biogenesis, manipulation, and potential applications of plant miRNAs in improving wheat’s yield, somatic embryogenesis, thermotolerance, and drought-tolerance in response to the problems of plateauing yield, genotype-dependent amenability to tissue culture, and susceptibility to climate change-induced heat and drought stress.

^©^ *His Majesty the King in Right of Canada, as represented by the Minister of Agriculture and Agri-Food, 2023.*

## 1 INTRODUCTION

Food security becomes more elusive as global agriculture struggles to keep up with the food demands of the rapidly increasing human population amidst the continuous reduction of agricultural lands, degradation of natural resources, and rapid climate change. Experts suggest a sustainable intensification of agricultural production to cope with the required annual crop productivity growth of 2-3% [1, 2]. This might prove difficult as modern crop varieties are bred to respond best to commercial production practices, some of which are perceived to cause harm to the environment. Moreover, while improved agronomic practices have afforded high productivity, the onset of extreme and unpredictable weather conditions consequent to climate change might increase the cost and reduce the effectiveness and applicability of modern agronomic practices and technologies. It is thus widely acknowledged that genetic gains through crop improvement will play a bigger role in pursuing future food security.

Wheat (*Triticum aestivum* L.) is one of the most important cereal crops, consumed by approximately 33 percent of the global population, and provides 19 and 21 percent of the daily carbohydrate and protein dietary requirements, respectively [3]. It is predominantly milled into wheat flour to make leavened bread, pasta, noodles, and other widely consumed baked products [4]. Moreover, aside from being the cheapest source of carbohydrates and protein, it also has an unparalleled range of cultivation and storability [5]. Unfortunately, wheat productivity has plateaued over the years, even registering the lowest rate of increase among major cereals [6]. Improving wheat productivity means furthering yield potential, minimizing crop losses, and increasing resource-use efficiency [2]. The urgent call to increase wheat productivity may not be met through conventional wheat breeding endeavours as the process takes a long time. The required diversity of genes and variations may not be present within wheat and its crossable relatives’ gene pools [7]. On the other hand, modern biotechnology's progress, especially genetic engineering, still remains limited in wheat as it is one of the most challenging crops to transform [8]. Wheat is highly recalcitrant to tissue culture. This is a problem since transformed plants are regenerated through tissue culture. Recently, Hayta *et al.* [8] developed a highly replicable *Agrobacterium*-mediated wheat transformation protocol with up to 77% transformation efficiency using a protein fusion of Growth Regulating Factor 4 (GRF4) and GRF Interacting Protein 1 (GIF1) discovered earlier by Debernardi *et al.* [9]. While this protocol’s success remains genotypically dependent, it dramatically improved the regeneration of transgenic wheat from tissue culture. Another difficulty in wheat transgenesis is the complexity of its genome. Wheat, an allohexaploid, has one of the biggest genomes, containing high amounts of gene redundancies and transposable elements [10]. These characteristics make identifying and manipulating novel protein-coding genes for wheat improvement difficult.

Recently, the use of microRNAs (miRNAs) as tools for gene studies and crop improvement has been gaining interest. These 20-24 nt endogenous, post-transcription repressors are highly specific to their target transcripts in plants [11] and are attractive targets for novel gene discoveries and genetic engineering since they regulate genes involved in almost all aspects of plant life, including but not limited to plant development, responses to biotic and abiotic stress, and plant architecture [12]. Furthermore, aside from the highly conserved nature of many miRNAs among plant species, the progress in understanding miRNA biogenesis and mode of action coupled with advances in bioinformatics and genomics allows for faster discovery of novel miRNAs and the subsequent prediction of their respective gene targets, furthering the development and popularity of miRNA-based crop improvement strategies.

Here, we will review miRNA biogenesis, the current tools for miRNA manipulations, and, most importantly, the potential applicability of miRNA technologies in combatting the challenges of wheat’s plateauing yield, genotype-dependent amenability to tissue culture, and susceptibility to climate change-induced heat and drought stress. Reviews exploring miRNA-mediated biotic stress responses are abundant [13, 14]. Therefore, this subject is excluded from this review.

### 1.1 miRNA Biogenesis and RNA-induced Silencing Complex (RISC) Formation

The exact mechanism of miRNA biogenesis in plants has yet to be elucidated. Comprehensive reviews were written by Achkar *et al.* (2016) [15] and Mencia *et al.* (2022) [16] regarding this topic, but for this review, the presented plant miRNA biogenesis will be simplified as illustrated in Fig. (**1**). Plant miRNAs are generally transcribed from intronic or intergenic MIRNA genes by a DNA-dependent RNA polymerase II (RNA Pol II) with the help of various transcription, mediator, and elongation protein complexes [17]. The resultant transcript, known as primary microRNA (pri-miRNA), shares the characteristics of other RNA Pol II products, specifically the 5´ cap and 3´ polyA tail. The limited self-complementarity of pri-miRNA transcript makes it assume a tailed stem-loop structure consisting of a loop, a stem, an miRNA/miRNA* duplex, a lower stem, and two tails [18]. While post-transcriptional and co-transcriptional pri-miRNA processing has been suggested [15, 16], this review will emphasize post-transcriptional processing. In this model, pri-miRNA is recruited into a Dicing Body (D-Body) where the microprocessor consisting mainly of the Dicer-like protein (DCL1), a zinc finger protein called Serrate (SE) and the RNA-binding Hyponastic Leaves (HYL1) protein co-localize [16, 18]. DCL1 manifests an RNase III activity, while SE and HYL mainly regulate the accuracy and efficiency of the dicing activity of DCL1 [15]. The concerted action of these three, along with other undefined microprocessor co-factors, remove the tails of the pri-miRNA to form the precursor miRNA (pre-miRNA), which is then cleaved in a base-to-loop or loop-to-base manner inside the D-Bodies, liberating miRNA-miRNA* duplex in complex with HYL1 [16]. The miRNA/miRNA* duplex contains 2 nucleotide 3´ end overhangs in each strand with 2´ OH and 3´ OH [18]. The 3´ end is methylated at the 2´ OH by the methyltransferase HUA Enhancer 1 (HEN1) to provide stability [19] and protection from SMALL RNA DEGRADING NUCLEASE [20]. Previous models suggested that the miRNA duplex’s export to the cytoplasm is facilitated by the HASTY (HST) protein, an EXPORTIN 5 homolog, but this has not been experimentally verified. On the contrary, the findings of Park *et al.* [21] demonstrated that single-stranded mature miRNAs can also be found within the nucleus and that, surprisingly, a loss of function mutation upon HST does not prevent miRNA accumulation in the cytoplasm. This suggests that the nucleus-loading of miRNA into ARGONAUTE1 (AGO1) also occurs and that there is an HST-independent export process. This is promoted by the findings of Cambiagno *et al.* [22], wherein a normal subcellular distribution of miRNAs was reported in *Arabidopsis thaliana hst* null mutants. The same study also reports that HST acts as a scaffold between DCL1 and MED37, a mediator complex subunit, suggesting that HST functions in miRNA biogenesis complex assembly instead of miRNA export. Furthering the idea that miRNA is loaded to AGO1 inside the nucleus was the discovery of highly conserved Nuclear Localization and Nuclear Export Signals on the N-termini of AGO1, suggesting that AGO1 is a nucleo-cytosolic shuttling protein and that miRNA-AGO1 loading predominantly occurs in the nucleus, facilitated by HYL and other proteins such as Heat Shock Protein 90 (HSP90) and/or Constitutive Alterations In The Small RNAs Pathway 9 (CARP9) [23, 24]. The AGO1:miRNA complex, or mature miRNA, gets exported from the nucleus into the cytoplasm through Nucleoporin (NUP1) embedded in the nuclear envelope [25]. In the cytoplasm, other proteins join the AGO1:miRNA to form the RISC, which represses target mRNAs through cleavage upon complete complementarity with the miRNA guide strand or translational repression upon partial complementarity [26]. The definite identity of proteins comprising the plant RISC is still unclear as of this writing.

### 1.2 miRNA Manipulation Strategies

Currently, plant miRNA-based strategies mainly revolve around the design of artificial miRNAs (amiRNAs) and the artificial modulation of miRNA expression levels (through overexpression or downregulation) as illustrated and summarized in Fig. (**2**). amiRNAs are produced by utilizing an endogenous pre-miRNA as a backbone but substituting the miRNA-miRNA* duplex with a sequence that is specific to the target gene’s mRNA in a manner that does not disrupt the original miRNA’s secondary structure [27]. Several bioinformatic tools are now available to design highly specific amiRNAs [28, 29]. amiRNAs are utilized in functional gene studies to silence genes of interest, producing ectopic phenotypes from which the gene functions can be inferred. It can also be used in crop improvement, wherein a gene of known function can be silenced to produce a desirable phenotype that is stable throughout future generations [27]. On the other hand, miRNA overexpression is achieved by utilizing a strong constitutive promoter upstream of the precursor miRNA sequence (pre-miRNA) and transforming them into plants. The strong constitutive promoter will allow greater transcription of pre-miRNA, resulting in a greater degree of target gene silencing/knockdown. miRNA overexpression is stable across generations and is thus meritorious for crop improvement. Lastly, miRNA downregulation, which aims to decrease miRNA expression, consequently upregulating the target gene’s activity, is achieved through target mimicry, transcriptional silencing of MIRNA gene promoters, and amiRNA-based anti-miRNA [30]. First demonstrated by Zorrilla *et al.* [31], target mimicry entails using mRNAs similar to miRNA targets but with mutations in the miRNA cleavage site. This results in miRNA sequestration as the target mimic binds to the RISC-guide miRNA but is not cleavable due to the mutations. On the other hand, transcriptional silencing of the MIRNA gene promoter prevents the access of the plant transcriptional machinery through repressive epigenetic marks on the *MIRNA* promoter. Hairpin RNA (hpRNA), designed to contain portions of genes or nucleotide sequences (in this case, *MIRNA* transcription start sites) in a sense-antisense direction [32] are expressed into the plant to trigger RNA-dependent DNA methylation, dramatically reducing pri-miRNA abundance [30]. Lastly, amiRNA-based anti-miRNA, demonstrated by Eamens *et al.* [33] to downregulate miRNA expression through post-transcriptional silencing, entails the substitution of the miRNA-miRNA* duplex with mature target miRNA sequence to silence the entire miRNA family, or with stem-loop sequence that is unique to a target miRNA for a miRNA-specific silencing.

### 1.3 miRNAs can be Targeted to Improve Somatic Embryogenesis

Somatic embryogenesis (SE), the process of regenerating embryos from somatic cells, is imperative to recovering genetically transformed plants. Unfortunately, wheat’s amenability for somatic embryogenesis remains generally poor and genotypically dependent [34]. As regulators of important biological processes, various miRNAs have been implicated in the SE of different economically essential crops [35] through the regulation of phytohormone signaling pathways [36]. Regarding the involvement of miRNAs in SE regulation, various studies have suggested that miRNAs are differentially expressed between embryogenic and non-embryogenic calli [37-40]. Moreover, it has also been reported that miRNA expression changes during the SE process in *A. thaliana* [41], *Zea mays* [42], *Gossypium hirsutum* [43], and *Citrus sinesis* [44]. Interestingly, manipulation of miRNA expressions has affected the SE capacities of various economically important crops as summarized in Table **1**. For instance, Shi *et al.* [45] have reported that overexpression of miR171 restored SE competence of recalcitrant *Citrus unshiu* cv. “Guoqing No.1’ calli in a dose-dependent manner. Moreover, it was also found that inhibition of miR171, through short tandem target mimic, weakened SE competence in the strongly embryogenic *Citrus sinesis* cv. “Valencia Sweet Orange” calli. Similarly, miR156, previously reported in rice to be involved in the transition toward differentiated calli along with miR397 [38], has recently been reported to be differentially expressed between embryogenic and non-embryogenic calli, and its overexpression has led to increased SE competence of *Fortunella hindsii* calli [46]. On the contrary, overexpression of miR167 in Arabidopsis has inhibited its SE capacity by altering auxin responsiveness and transport through downregulating its targets: AUXIN RESPONSE FACTOR 6 and 8 (*AtARF6* and *AtARF8*) [47]. Yao *et al.* [48] had previously identified these miRNAs to be conserved in wheat, suggesting that their expression can also be modulated to increase SE success in the highly recalcitrant elite genotypes.

### 1.4 miRNAs can be Used to Increase Wheat’s Yield Potential

Sustainably increasing crop productivity per unit of land is touted as the solution to feeding the ever-growing human population amidst continuously declining agricultural land. However, yield is a highly complex multigenic trait that needs to be fully understood. Even modern advances in genetic engineering have yet to significantly impact the production of crop varieties with high-yielding potential [49]. Recent studies have, however, demonstrated that over-expression or repression of specific conserved miRNAs produced crop lines with greater yield relative to wild type (Table **1**) Overexpression of miR408, a widely conserved miRNA in both monocots and dicots, has resulted in a significant yield increase in *Oryza sativa* [50] and *Arabidopsis thaliana* [51] by affecting copper homeostasis in favor of plastocyanin. This blue copper protein acts as a mobile electron acceptor for photosynthesis. By downregulating the transcripts of its target genes UCLACYANIN 8 (*OsUCL8*), *LACCASE 13* (*AtLAC13*), *PLANTACYANIN* (*AtARPN*), miR408 increases plastocyanin levels, elevating the electron transport rate and photosynthetic efficiency, resulting in higher grain yield. Aside from yield increase, these studies also report that miR408 overexpressing plants have a taller phenotype with more biomass relative to wildtype, indicating that this miRNA also targets genes related to rice and *Arabidopsis thaliana* morphology. Unfortunately, a taller phenotype may cause yield losses due to lodging, especially along with indiscriminate fertilizer applications. The presence of miR408 in wheat has been verified by Yao *et al.* [48], and its overexpression has resulted in earlier and shorter heading times [52].

Similarly, overexpression of OsamiR397 in *Oryza sativa* has been reported by Zhang *et al.* [53] to increase grain size and panicle branching, thereby increasing yield by up to 25% in field trials. Osmi397 has been reported to target the transcripts of the LACCASE (*OsLAC*) genes responsible for brassinosteroid sensitivity. This was demonstrated by OsmiR397-overexpressing rice plants becoming more sensitive and responsive to brassinosteroid treatments, while the *OsLAC-*overexpressing plants showed the opposite [53]. Yao *et al.* [48] reported the presence and low expression of miR397 in wheat, while Gupta *et al.* [54] reported that the said miRNA was downregulated during osmotic, salt, and cold stress. On the other hand, the sequestration of miR396b through target mimicry was reported by Gao *et al*. [55] to increase rice yields by altering inflorescence architecture. miR396b directly targets the Growth Regulating Factor 6 (*OsGRF6*) gene. By overexpressing the artificial miR396b target mimic, the downregulation of *OsGRF6* was significantly impaired, resulting in its elevated expression, increased spikelet number, and yield. Li *et al.* [56] reported that miR396 was one of the conserved miRNAs involved in wheat grain development.

### 1.5 miRNAs are Associated with Heat Response and Thermotolerance

With rising global temperatures consequent to climate change, conferring thermotolerance to wheat becomes more urgent. In wheat, heat stress can adversely affect the reproductive stage and grain filling [57], causing pollen sterility, drying of stigmatic fluid, pseudo-seed setting, empty pockets in the endosperm, and shriveled seeds [58], all of which lead to significant yield losses. Kumar *et al*. [58] reported that wheat utilizes heat-responsive miRNAs as a defense mechanism against heat stress. Indeed, plenty of miRNA families have been said to be functional in heat stress responses such as reactive oxygen species (ROS) scavenging, heat shock proteins (HSPs) activation, photosynthetic systems protection, reproductive systems protection, and biogenesis of other miRNAs [59]. Indeed, differentially expressed miRNAs in wheat were identified and validated by Kumar *et al.* [60] and Ravichandran *et al.* [61] upon heat stress induction. For instance, miR156, a highly conserved miRNA reported to be heat responsive in wheat, was associated with heat stress memory in *Arabidopsis* [62]. They further revealed that elevated expression of AtmiR156 can promote sustained expression of heat-responsive genes, resulting in the continued maintenance of acquired thermotolerance during heat stress. Overexpression of miR156, and the consequent downregulation of its target, the squamosa promoter-binding protein-like (*SPL*) genes, were found to substantially improve the thermotolerance of Alfalfa to heat stress of 40°C, with the transgenic plants manifesting higher water potential, and non-enzymatic antioxidant, anthocyanin, and chlorophyll content [63]. Another highly conserved miRNA reported by Ravichandran *et al.* [61] to be heat-responsive in wheat was miR398. Targeting copper/zinc superoxide dismutase 1, 2 (CSD1, CSD2) and copper chaperone for superoxide dismutase (CCS), this miRNA was reported to be directly induced by the heat-responsive *HEAT SHOCK TRANSCRIPTION FACTOR HSFA1b and HSFA7b* in *Arabidopsis* [64]. In the same study, it was also reported that the null mutants of miR398 targets showed better heat stress response, notably less flower damage, relative to wild type and mutants with miR398-resistant target genes. A summary of these miRNAs, the tools used for manipulating their expressions, and the resulting phenotype is presented in Table **1**.

### 1.6 miRNAs are Involved in Water-stress Response

As water scarcity is exacerbated by climate change, water stress becomes an increasingly important wheat production hurdle. Historical worldwide data has shown that at approximately 40% water reduction, wheat yield decreased by 20.6% [65]. Exposure to water stress at critical developmental stages, specifically during stem elongation and heading steps, resulted in substantive yield penalties [66]. As regulators of important biological processes, miRNAs have been implicated in plant responses to water stress. For instance, the overexpression of miR408 has been reported to induce tolerance to drought, an extreme form of water stress, in *Cicer arietinum* [67, 68] and *Lolium perenne* [69]. Moreover, it was also reported that miR408 was differentially expressed in drought-tolerant and susceptible rice varieties upon exposure to drought stress, wherein the transcript levels of the said miRNA remained high in tolerant varieties but were substantially downregulated in sensitive varieties [70, 71]. A similar contrasting expression of miR408 was reported between the drought-tolerant wild *Ipomea campanulata* and *Jacquemontia pentantha* upon induction of water stress [72]. In *Cicer arietinum*, a closer investigation by Hajyzadeh *et al.* [67] revealed that overexpression of miR408 instigated a cascade of reactions that also regulated several drought-responsive genes such as Dehydration Responsive Element Binding Protein-Responsive To Dehydration (DREB-RD modules) and Basic Helix-Loop-Helix 23 (BHLH23), both were previously identified to be responsive to copper levels [73, 74]. Interestingly, in wheat, miR408 was upregulated in leaves upon drought stress exposure [75] in drought-tolerant and susceptible varieties [76].

Bakhshi *et al.* [76] reported more differentially expressed miRNA between drought-tolerant and susceptible wheat cultivars upon drought exposure. Among these, miR166 was found to be upregulated in the tolerant variety while downregulated in the susceptible variety. Interestingly, overexpression of this miRNA has been linked to improved drought stress responses, including higher relative water content, lower electrolyte leakage, and better rates of photosynthesis, transpiration, and water use, when done in parallel to the inoculation of Plant Growth Promoting Rhizobacteria *Pseudomonas putida-RA* [77]. On the contrary, Zhang *et al.* [78] reported that the knockdown of Osa-miR166 led to increased drought tolerance in rice through decreased stomatal conductance, transpiration rate, and reduced hydraulic activity of the xylem. These mutant plants also manifested a rolled-leaf phenotype, a typical drought stress response, by default. Lastly, a drought-responsive wheat miRNA, TaemiR1119, was reported to be upregulated in roots upon drought stress treatment [79]. According to the researchers, overexpressing this miRNA in *N. benthamiana* resulted in significantly higher plant biomass, photosynthetic parameters, osmolyte accumulation, and antioxidant enzyme (AE) activities than wild-type ones. Table **1** summarizes the effects of modulating these water-stress responsive miRNAs to the water-stress tolerance of different plant species.

## CONCLUSION

Wheat is a widely grown and consumed crop vital to the pursuit of food security. Unfortunately, due to its large and highly complex hexaploid genome, identification and functional validation of novel genes for wheat improvement are difficult. Wheat production is currently faced with a plateauing yield expected to worsen due to elevated global temperatures and water scarcity associated with climate change. Moreover, the impact of biotechnology in addressing these challenges remains unmaximized due to wheat’s genotypically-dependent amenability to tissue culture regeneration. This review explored the prospect of addressing these challenges through miRNA technologies. miRNAs are one of the primary regulators of biologically essential processes and, by extension, agronomically important traits. These 20-24 nucleotide repressors can target multiple genes, hence holding unrealized potential for improvement of complex, multigenic traits such as yield and stress responses. Most importantly, with continuous improvements in bioinformatic computational capacity, coupled with the widely conserved nature of plant miRNAs, gene targets, and consequently, miRNA functions, can be reasonably predicted and validated through various assays such as transient co-expression assays of a miRNA and its putative targets, or modulation, either overexpression or knockdown, of a miRNA or its putative targets.

## Figures and Tables

**Fig. (1):**
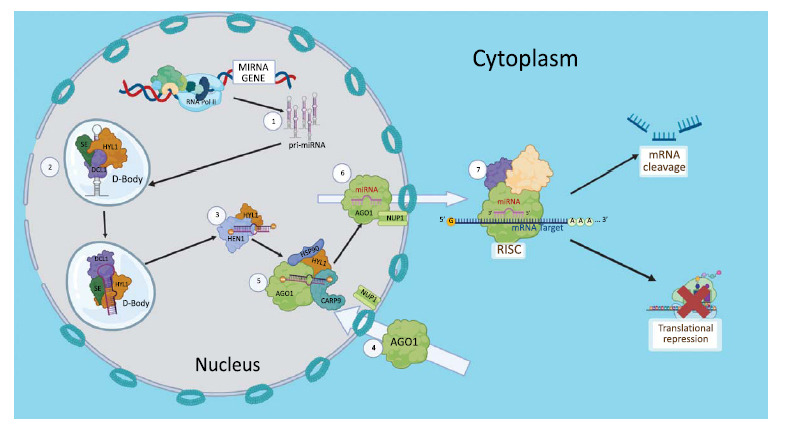
Diagram of a simplified miRNA biogenesis pathway in plants. The limited self-complementarity of MIRNA gene transcripts facilitates the formation of a stem-loop structure called pri-miRNA [1], which localizes inside D-Bodies where it is cleaved by a microprocessor consisting mainly of serrate (SE), dicer-like protein (DCL1), and hyponastic leaves (HYL1) [2]. After processing, a miRNA-miRNA* duplex in a complex with HYL1 is released and methylated by HUA enhancer 1 (HEN1) [3]. The argonaute1 (AGO1) shuttles inside the nucleus with the help of nucleoporin (NUP1) [4], where it is loaded with the correct strand of mature miRNA through the help of other proteins such as Hyl, heat shock protein 90 (HSP90), and constitutive alterations in the small RNAs pathway 9 (CARP9) [5]. The AGO1:miRNA ribonucleoprotein gets exported into the cytoplasm with the help of NUP1 [6], where it complexes with other proteins to form the RNA-induced silencing complex (RISC), executing cleavage or translational inhibition of target mRNAs [7].

**Fig. (2):**
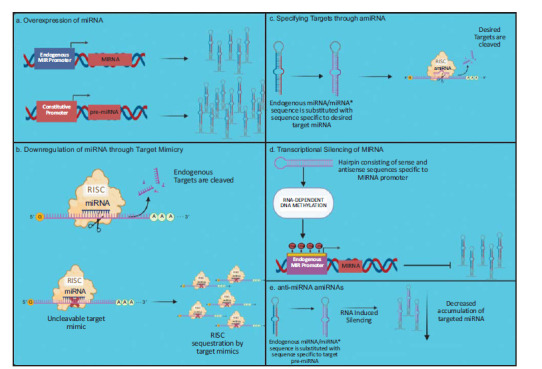
Diagram of various miRNA manipulation strategies. **a**) The pre-miRNA sequence is placed downstream of a strong constitutive promoter to elevate its expression level. **b**) Uncleavable target-mimic transcripts of a specific miRNA sequester RISC, thus lowering its silencing activity. **c**) A sequence specific to the gene of interest replaces the endogenous pre-miRNA nucleotide sequence at the miRNA/miRNA* duplex. The RISC, now guided by the amiRNA, will then silence the gene of interest. **d**) Introducing a hairpin structure consisting of a sense/antisense sequence specific to the endogenous MIRNA promoter triggers an RNA-dependent DNA methylation of the MIRNA promoter region. **e**) An endogenous pre-miRNA nucleotide sequence is replaced at the miRNA/miRNA* duplex by a sequence specific to the target miRNA’s precursor sequence, silencing the target miRNA.

**Table 1:** Summary of miRNAs that are conserved in wheat and their effects on other plant species upon manipulation.

**Plant Species**	**miRNA**	**Manipulation Strategy**	**Resulting Phenotype**	**References**
*Citrus unshiu* cv. “Guoqing No.1	CsimiR171c	Overexpression through *CaMV 35s* promoter	Increased somatic embryogenesis competence of previously recalcitrant calli	[45]
*Citrus sinesis* cv. “Valencia Sweet Orange”	CsimiR171c	Downregulation through short tandem target mimic	Abolished somatic embryogenesis of previously amenable calli	[45]
*Fortunella hindsii*	CsimiR156a	Overexpression through *CaMV 35s* promoter	Increased somatic embryogenesis competence of previously recalcitrant calli	[46]
*Arabidopsis thaliana*	AthmiR167c	Overexpression through *CaMV 35s* promoter	Induced somatic embryogenesis	[47]
*Oryza sativa*	OsamiR408	Overexpression through *CaMV 35s* promoter	Increased yield, biomass, height, and photosynthetic efficiency	[50]
*Arabidopsis thaliana*	AthmiR408	Overexpression through *CaMV 35s* promoter	Increased yield, biomass, silique length, and photosynthetic efficiency	[51]
*Oryza sativa*	OsamiR397	Overexpression through *CaMV 35s* promoter	Increased grain size and panicle branching	[53]
*Oryza sativa*	OsamiR396	Downregulation through target mimicry	Increased grain yield through modified inflorescence	[55]
*Arabidopsis thaliana*	AthmiR156	Overexpression through *CaMV 35s* promoter	Longer duration of acquired thermotolerance and heat stress memory; higher leaf initiation and delayed flowering	[62]
*Medicago sativa*	MsamiR156	Overexpression through *CaMV 35s* promoter	Improved heat tolerance	[63]
*Cicer arietinum*	AthmiR408	Overexpression through *CaMV 35s* promoter	Improved drought tolerance	[67]
*Cicer arietinum*	Vunmir408	Overexpression through *CaMV 35s* promoter	Enhanced drought and salt tolerance; increased trichome density and lower stomatal aperture	[68]
*Lolium perenne*	OsamiR408	Overexpression through *CaMV 35s* promoter	Enhanced drought tolerance and antioxidant capacity, sunken stomata, leaf curling	[69]
*Arabidopsis thaliana*	CarmiR166	Overexpression through *CaMV 35s* promoter and inoculation with *Pseudomonas putida*, RA strain	Enhanced germination upon drought, improved drought tolerance, less membrane damage	[77]
*Oryza sativa*	OsamiR166	Downregulation through short tandem target mimic	Rolled leaves, smaller leaf bulliform cells, altered xylem vessel, enhanced drought tolerance	[78]
*Nicotiana benthamiana*	TaemiR1119	Overexpression through *CaMV 35s* promoter	Improved biomass, photosynthesis, osmolyte accumulation, and anti-oxidant while faced with drought stress	[79]

## LIST OF ABBREVIATIONS

AE: Antioxidant Enzyme
AGO1: Argonaute1
amiRNAs: artificial miRNAs
BHLH23: Basic Helix-loop-helix 23
CARP9: Constitutive Alterations in the Small Rnas Pathway 9
CCS: Copper Chaperone for Superoxide Dismutase
CSD1, CSD2: Copper/zinc Superoxide Dismutase 1, 2
D-Body: Dicing Body
DREB-RD: Dehydration Responsive Element Binding Protein-Responsive to Dehydration
GRF4: Growth Regulating Factor 4
HEN1: HUA Enhancer 1
hpRNA: Hairpin RNA
HSP90: Heat Shock Protein 90
HSPs: Heat Shock Proteins
HYL1: Hyponastic Leaves
miRNAs: microRNAs
NUP1: Nucleoporin
OsGRF6: Growth Regulating Factor 6
pri-miRNA: Primary microRNA
RISC: RNA-induced silencing complex
ROS: Reactive Oxygen Species
SE: Somatic Embryogenesis
